# Bilateral Staged Total Hip Replacement and the Natural Progress of an Untreated Case of Developmental Dysplasia (Dislocation) of the Hip: A Clinical Case Report by the Surgeon and the Patient

**Published:** 2015-07

**Authors:** Hamid Honarpisheh, Mohammad Taghi Ghazavi

**Affiliations:** 1 Iranian Medical Science Councils Secretariat, Deputy of Education, Ministry of Health and Medical Education, Tehran, Iran;; 2 Orthopedic Surgery, Shafa Orthopedic Hospital, Iran University of Medical Science, Tehran, Iran

**Keywords:** Hip dysplasia, Total hip replacement, Comorbidity, Developmental dysplasia of the hip

## Abstract

The natural history of an untreated case of a Developmental Dysplasia (Dislocation) of the Hip (DDH) associated with multiple congenital abnormalities is reported in a 55-years-old man. The patient’s complaints and the varieties of the typical manifestations emerged in other parts of the body throughout the life are reviewed and discussed as comorbidities of a dysplastic condition. Two-stage bilateral total hip replacement (THR) operations were performed at the age of 55. In addition, to relieve the pain, the walking disabilities were overcome, hence gaining normal walking in swing and stances. The leg length discrepancy was corrected by anatomically positioned prostheses, examined by the knee bending test and characterized and evidenced by radiological features and indices.

## Introduction

Hip dysplasia or DDH is a congenital and developmental malformation of the hip joint. If untreated, bilateral hip joint osteoarthritis is unavoidable. It may be unilateral or bilateral. Patients referred with seemingly unilateral developmental dysplasia are also at the risk of having anticipated contralateral secondary problems in future.^1^ Although it is concluded that late diagnosis of DDH is far greater than those reported in some studies, it is suggested that to avoid over-diagnosis in the first week examination, repeated clinical and sonographic evaluations are required.^2^^,^^3^ Together with the hormonal link, specifically relaxin is suggested to be involved in DDH.^4^ Some genetic and familial factors are also indicated in DDH cases by the trait running in families.^5^

The results of various methods for obtaining and assessing comorbidity information for patients undergoing joint replacement as reported^6^ may be mixed up with the complications of the operation. Total hip arthroplasty (THA) in high riding congenital dislocation of the hip is a challenging procedure. To position the cup of the prosthesis in the true acetabulum, femoral shortening osteotomy is often considered to prevent nerve damage. The results of two different methods of femoral shortening osteotomy evaluated and considered in other studies, may not be applicable in this and many other cases. Either proximal or distal femoral shortening osteotomy could have different advantages and disadvantages per se and in different conditions. Proximal shortening osteotomy is concluded to be a more challenging procedure, may need special stem design, and could compromise stem fixation.^7^ However, the experience of surgeon drastically affect the outcome.

## Case Report

Staged bilateral total hip arthroplasties were performed on a 55-year-old man with a neglected untreated history of DDH, who gradually developed severe bilateral hip joint arthritis. The chief complains included bilateral hips and knee joints pain on walking associated with a progressive limitation in the range of hip motion and walking ability distance day after day. The history of the present problem went back to the age 45 with an incident of the low back pain repeated at the ages 50 and 52; each time relieved by conventional bed rest and other conservative measures. The progressive changes in lumbar spine (L2-L3) including discopathies and stenosis of the spinal canal were also contributed to his limitation of walking ability for last 10 years.

The patient was born as the last member of a large size family of a second degree consanguineous traditional marriage, with the maternal age 43, having 3 sisters and 4 brothers without hip disease. In the past medical history, the patient presented a history of the right nasolacrimal stenosis operated at the age of 3 and 8, right inguinal hernia operated for at 1 and 3. A suspected dentine dysplasia characterized by deciduous and permanent teeth with clinically normal appearing crowns, some hypermobility and spontaneous dental abscesses started in childhood and ended with the loss of all teeth at the age of 22 to 27. An unsuccessful closed reduction of the left hip joint at the age of 11 put in spica cast from the tip of the toe up to the chest level was performed and immobilized for 9 months. A history of night bedwetting, up to the age of 11, due to urinary reflux evolving in the left renal regression and hypoplasia, accidentally revealed upon a renal infection and diagnosed by renal angiography at the age of 22. A problem of ingrown toe nail started from the age of adolescence with multiple recurrence after a surgical correction was noticed. Two attacks of the left ear Menier’s disease reported to be experienced at the age of 33 and 44 years, ended with the total hearing loss and a residual continuous tinnitus. The patient and family had a habit of W-sitting from the childhood. The patient had no history of smoking or alcohol use. NSAIDS was the only medication he received. He was married having two children, a daughter (16 years old) and a son (15 years old), none of them having hip problems.

Physical examination revealed marked limitation of motion with some pelvic obliquity, a positive bilateral Trendelenberg test, mild gluteal asymmetry, apparent limb length discrepancy, severe crepitation during hip flexion, and painful hip in long time standing and walking.

**Figure 1:**
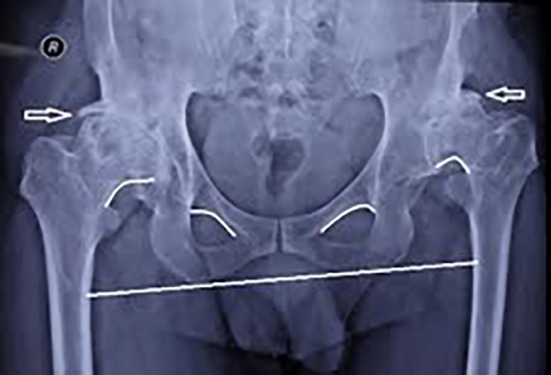
Pelvic X-ray view showing neolimbi and diagnostic landmarks.

**Figure 2:**
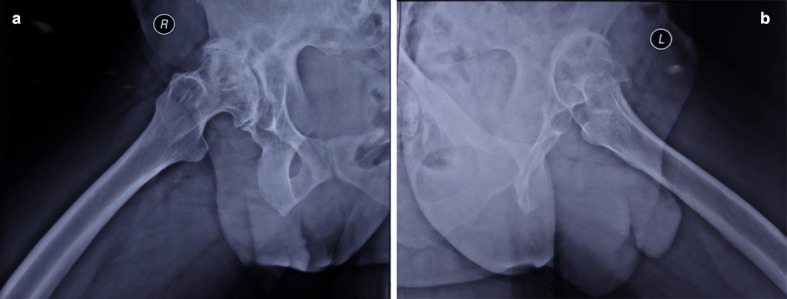
a) Right hip X-ray lateral view, b) Left hip X-ray lateral view.

The pelvic X-ray showed false acetabulum, broken Shenton’s line which was worse on the left side, severe arthritis changes such as diminished joint space, ossified neolimbi on both sides as shown in figure 1 (arrows), large osteophytes and bony spurs, loss of cartilage, massive sclerotic changes on both sides, dislocation on the left and subluxation on the right side. Increased anteversion of the femoral neck of the left side and lateral views are shown in lateral views of both hips (figures 2a and 2b). 

**Figure 3:**
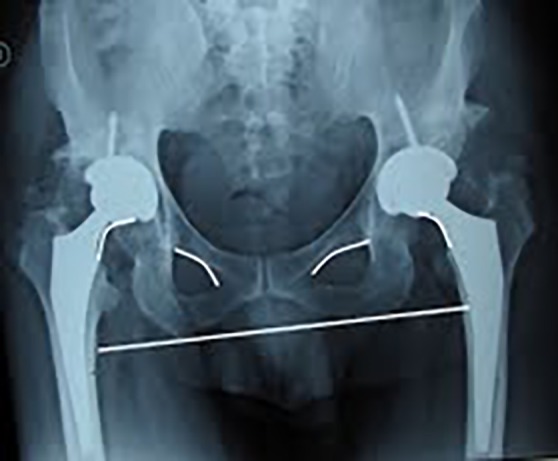
Post-op bilateral pelvic X-ray showing the equally spaced broken Shenton’s lines.

Uneventful cementless total hip arthroplasties were performed on both hips. The acetabular cups were placed close to anatomic true acetabulum. Post-op anteroposterior view of the pelvis shows implantation of the femoral and acetabular components (Titanium cementless press fit). The movement occurs between a chrom-cobalt head on a highly cross linked polyethene liner (figure 3).

**Figure 4:**
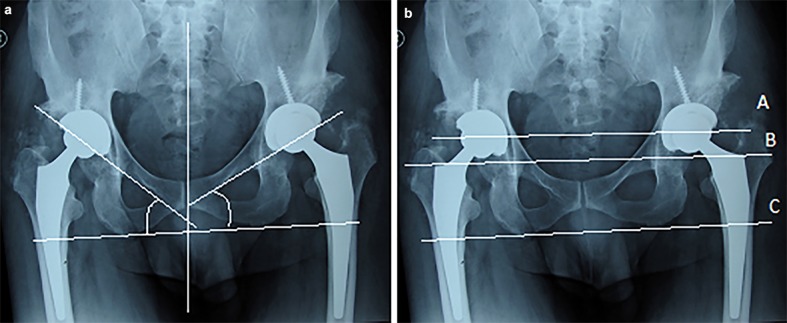
THR post-op bilateral pelvic radiographs; the left figure a) illustrates the lateral inclination of acetabular components and the right figure, b) illustrates the vertical assessment of femoral components.

The patient got ambulated walking with crutches the day after the surgery and was able to walk without crutches six weeks after the second operation. During the first six week post-op, conventional physical therapy was instructed. No leg length discrepancy was noticed seven days after the second THR operation and remained as such thereafter (table 1). This is demonstrated by the symmetrically located stem part of the prostheses in relation to the pubic ramus verified by equally broken Shentons’ lines shown in the post-op pelvic radiograph (figure 4).

**Table 1:** The results of one leg knee bending test while standing on the other leg indicating the leg length discrepancies

**Time (days)**	**Maximum number of steps walking in a day**	**Weight bearing distribution while standing by walker/crutches**			**One leg knee bending while standing on both legs/the other leg, indicating the legs length discrepancies**				
		**Both hands**	**Left leg (newly operated)**	**Right leg**	**Maximal (>25 degrees)**	**About 20-25 degrees**	**About 10-20 degrees**	**Minimal** **(<5 degrees)**	**No**
Before operation	<200	10-20%	30%	50%	X				
Operation day	0								
Day 1-2 post-op	4	>90%	0%	10%	X				
Days 3-4 post-op	12	>80%	5%	10%		X			
Day 5-6 post-op	50-96	>60%	10%	20%			X		
1^st^ to 2^nd^ week post-op	120	<60%- <40%	20%	20%					X
3^rd^ week	200	<20%	30%-50%	50%-30%					X
4^th^ week	200	0%*	<10%->90%	>90%-<10%					X
6^th^ week	>1000	0%*	50%	50%					X

*Indicates using no crutches

## Discussion

Two questions are discussed for didactic purposes in this case. The first question of etiopathogenic concern investigated is that: could dysplasia be considered as the one common predisposing developmental or congenital factor for the comorbidities manifested throughout the life in this case? Poorly developed acetabulum is observed and evidenced on the left side (figure 1). Bilaterally developed false acetabulum causing increased degenerative changes, elongated capsules covering the femoral heads, and lumbar spine disc problems associated with the above mentioned complains, as well as the functional disabilities evolved at the age of 55 in such a male patient signifies the thorough natural history of a typical developmental dysplastic hip dislocation.^8^^,^^9^ Similar cases are reported as isolated clinical problem presentation. What is differently hypothesized is that the natural history of the developmental dysplastic disease of the hip with the evidences of other tissue dysplasia may be due to some common genetic factors in this case. As in this case, it includes dysplastic changes in the tendons and cartilaginous structures of the abdominal wall causing recurrent abdominal hernia, detrusor muscle of the bladder involved in night refluxes, the inner ear underlying Menier’s disease of the left ear, a suspected dentine dysplasia associated with dental abscesses in the past medical history of the patient,^10^^,^^11^ the vertebral discopathy and the ingrowing toe nails matrices. Further, it seems that the dysplastic process can be not limited to the embryonic, fetal, infantile periods or even years of childhood, but in such a full blown case, the process may penetrate and continue up to the ages of 55. The conditions, life style, and physical activities may affect the time of the emergence of each manifestation in this process, presenting the developmental aspects of this dysplastic condition in different parts of the body.

The second question is of therapeutic concern and seeks the crucial specific reconstructive conceptual approach in the THR operations that may be proposed to best compensate for the discrepancy in the leg length after the first operation.^7^^,^^12^ A somehow asymmetric THA plan would rationally compensate for the discrepancy in the leg length.

Other than the equally broken Shentons’ line, findings shown in the post-op pelvic radiograph mentioned previously, the following radiographic indices are analyzed to discuss the mechanism of compensating for the leg length discrepancy after the second operation. Generally the angle of acetabular index, inclination or opening of the acetabulum should normally be about 30-50º (figure 4a). The distance from the center of each femoral head to the lines that intersect the ischial tuberosities (between lines A and C) and to the greater trochanters (between lines B and C) should be symmetric^13^ (figure 4b). In this case, it is so positioned in reference to the prosthesis lateral notch as to compensate for the leg length discrepancy and the results of the differential knee bending test, mentioned above, shows that it worked well (figure 4b).

This is a typical lifelong lesson as a lesson for lifelong learning and an important valid way of need assessment in medical education that mainly rely on the Patients’ complaints and feedback, adverse events, patient satisfaction report and the knowledge of patient sources that cannot be replaced by other sources. Different learning methods tend to suit different doctors and differently identified learning needs. Although physicians are supposed to use a wide variety of formal and informal ways of identifying their own learning needs, as part of their ordinary practice, none of those methods would substitute for patients’ experiences and their chief complaint. *Patient self-reporting* may be considered an alternative to chart review and discharge diagnosis, since the patient is the primary source of information.^14^

## Conclusion

In this case, we tried to reflect on possible emergence of different manifestations of the dysplastic process presenting the developmental aspects of this condition in different parts of the body throughout the life. In the total hip replacement operations, the total hip prostheses are asymmetrically positioned to compensate for the leg length discrepancy and the results of the differential knee bending test, mentioned above, shows that it worked well. We strongly recommend the physicians to report their case no matter how common and typical they seem to be, because all the feelings and impressions they report related to their illness reflect real experiential learning values. 
